# Identification of the *Staphylococcus aureus* MSCRAMM clumping factor B (ClfB) binding site in the *α*C-domain of human fibrinogen

**DOI:** 10.1099/mic.0.2007/010868-0

**Published:** 2008-02

**Authors:** Evelyn J. Walsh, Helen Miajlovic, Oleg V. Gorkun, Timothy J. Foster

**Affiliations:** 1 Microbiology Department, Moyne Institute of Preventive Medicine, Trinity College, Dublin 2, Ireland; 2 Department of Pathology and Laboratory Medicine, CB #7525, Brinkhous-Bullitt Building, University of North Carolina at Chapel Hill, Chapel Hill, NC 27599-7525, USA

## Abstract

Clumping factor B (ClfB) of *Staphylococcus aureus* binds to cytokeratin 10 and to fibrinogen. In this study the binding site in human fibrinogen was localized to a short region within the C terminus of the A*α*-chain. ClfB only bound to the A*α*-chain of fibrinogen in a ligand-affinity blot and in solid-phase assays with purified recombinant fibrinogen chains. A variant of fibrinogen with wild-type B*β*- and *γ*-chains but with a deletion that lacked the C-terminal residues from 252–610 of the A*α*-chain did not support adherence of *S. aureus* Newman expressing ClfB. A series of truncated mutants of the recombinant A*α*-chain were tested for their ability to support adherence of *S. aureus* Newman ClfB^+^, which allowed the binding site to be localized to a short segment of the unfolded flexible repeated sequence within the C terminus of the A*α*-chain. This was confirmed by two amino acid substititions within repeat 5 of the recombinant A*α*-chain which did not support adherence of Newman ClfB^+^. *Lactococcus lactis* expressing ClfB mutants with amino acid substitutions (N256 and Q235) located in the putative ligand-binding trench between domains N2 and N3 of the A-domain were defective in adherence to immobilized fibrinogen and cytokeratin 10, suggesting that both ligands bind to the same or overlapping regions.

## INTRODUCTION

*Staphylococcus aureus* is an important opportunistic pathogen of humans that is responsible for a wide range of infections ranging from superficial skin infections to more serious invasive diseases such as endocarditis, osteomyelitis and septicaemia. The primary habitat of *S. aureus* is the moist squamous epithelium of the anterior nares (Cole *et al.*, 2001; Peacock *et al.*, 2001). The success of *S. aureus* as a pathogen is due in part to its ability to adhere to a wide range of host tissues including host extracellular matrix proteins such as fibrinogen (Fg), fibronectin and collagen. Adhesion to host proteins is mediated by bacterial cell-wall-associated proteins called MSCRAMMs (microbial surface components recognizing adhesive matrix molecules). *S. aureus* can express up to 20 different potential MSCRAMMs that are covalently anchored by sortase to peptidoglycan (Mazmanian *et al.*, 1999; Navarre & Schneewind, 1994).

*S. aureus* expresses several different proteins that can bind specifically to Fg, including clumping factors A and B (ClfA and ClfB) and the bifunctional fibronectin- (and Fg-) binding proteins A and B, FnbpA and FnbpB (McDevitt *et al.*, 1994; Ni Eidhin *et al.*, 1998; Perkins *et al.*, 2001; Wann *et al.*, 2000). ClfA, FnbpA and FnbpB bind to the extreme C terminus of the *γ*-chain protruding from domain D of Fg. In contrast, the SdrG protein from *Staphylococcus epidermidis* binds to the fibrinopeptide-B protruding from domain E (Davis *et al.*, 2001). *S. aureus* also secretes several proteins that bind Fg, notably coagulase, the extracellular Fg-binding protein (Efb) and MHC class II analogue protein (Map) (Jonsson *et al.*, 1995; McGavin *et al.*, 1993; Palma *et al.*, 1998; Phonimdaeng *et al.*, 1990).

ClfB is only expressed on the cell surface during the exponential phase of growth (McAleese *et al.*, 2001). Ligand binding is specified by the A region, which is divided into three independently folded subdomains N1, N2 and N3 (Perkins *et al.*, 2001). The *S. aureus* metalloprotease aureolysin cleaves ClfB between N1 and N2, resulting in the loss of Fg-binding activity at the end of the exponential phase of growth (McAleese *et al.*, 2001; Perkins *et al.*, 2001). ClfB is a bifunctional MSCRAMM. It binds to cytokeratin 10 (CK10) exposed on the surface of desquamated epithelial cells in addition to Fg. It is a major determinant of the ability of *S. aureus* to adhere to squamous cells and to colonize the anterior nares (O'Brien *et al.*, 2002). The binding domain in CK10 was shown to be quasi-repeats of glycine and serine residues that occur as unfolded loops located at the C terminus of the protein, which likely protrude from keratin filaments (Walsh *et al.*, 2004).

**Fig. 1. f1:**
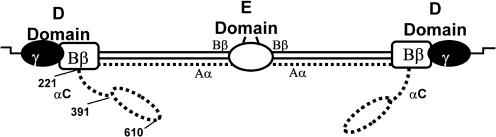
Structure of fibrinogen. Fg consists of two identical disulfide-linked subunits, each of which is composed of three non-identical polypeptide chains, Α*α*, B*β* and *γ*. Fg can be divided into four major regions, the central E region, two identical terminal D regions and the *α*C-domains. The *α*C-domains (residues 221–610) contain two distinct regions, a compact C-terminal half and an unordered NH_2_-terminal half or connector region.

Fg is a 340 kDa plasma protein that plays a crucial role in haemostasis. It is composed of two identical disulfide-linked subunits, each of which is composed of three non-identical polypeptide chains, Α*α*, B*β* and *γ* (Doolittle, 1984; Henschen & MCDonagh, 1986; Herrick *et al.*, 1999). The removal of C termini of the A*α*-chains (residues 220–610) by proteolysis results in generation of *α*C fragments, representing the whole or parts of the *α*C-domain (Weisel & Medved, 2001). The *α*C-domains are involved in fibrin assembly and clot formation (Cierniewski & Budzynski, 1992; Gorkun *et al.*, 1994; Medved *et al.*, 1985) and control activation of factor XIII (Credo *et al.*, 1981). The *α*C-domains and the N-terminal portions of the B*β*-chains are the parts of the Fg molecule for which the 3D structure has not been established (Yang *et al.*, 2001). However, recent studies have shown that the *α*C-domain consists of two structurally distinct regions, a flexible connector region from residues 221–391 and an independently folded, compact portion from residues 392–610 (Fig. 1) (Burton *et al.*, 2006; Tsurupa *et al.*, 2002). In human Fg the flexible NH_2_-connector region is unordered and is composed of a 43-residue segment followed by ten 13-residue tandem repeats.

In this study we investigated the binding of ClfB to human Fg and have localized the binding site to one of the tandem repeats within the flexible connector region of the *α*C-domain.

## METHODS

### Bacterial strains and growth conditions.

*Escherichia coli* strain XL-1 Blue was used as the host for plasmid cloning and was routinely grown in L-broth or agar with ampicillin (100 μg ml^−1^) and tetracycline (10 μg ml^−1^) as appropriate. Strain TOPP3 (Stratagene) was used as the host for recombinant ClfB A-domain (rClfB 45–542) or rClfA A-domain (rClfA 221–559) protein expression. *E. coli* strain JM101 was used for expression of recombinant Fg proteins.

The *S. aureus* strains are mutants of strain Newman (Duthie & Lorenz, 1952) defective in ClfA (DU5876 *clfA2* : : Tn*917*) (McDevitt *et al.*, 1994) and a double mutant defective in ClfA and ClfB (DU5944 *clfA2* : : Tn*917 clfB* : : Tc^r^) (Ni Eidhin *et al.*, 1998). Bacteria were routinely cultured in trypticase soy broth or on agar. For optimum expression of ClfB, *S. aureus* was grown to exponential phase (OD_600_ 0.6) in 50 ml brain heart infusion broth in a 250 ml conical flask shaken at 200 r.p.m. at 37 °C (McAleese *et al.*, 2001; Ni Eidhin *et al.*, 1998).

*Lactococcus lactis* strain NZ9800 carrying the nisin-inducible expression plasmid pNZ8037 expressing *clfB* or *clfB* Q235A was described previously (Miajlovic *et al.*, 2007). *L. lactis* pNZ8037*clfB* N526A was constructed by site-directed mutagenesis as described for *L. lactis* pNZ8037*clfB* Q235A (Miajlovic *et al.*, 2007) using the primers shown in Table 1. *L. lactis* strains were grown statically at 28 °C in M17 (Difco) broth incorporating 0.5 % (w/v) glucose, chloramphenicol (Sigma, 10 μg ml^−1^) with nisin at 3.2 ng ml^−1^ to stimulate maximum induction (Miajlovic *et al.*, 2007).

### Manipulation of DNA.

Restriction and DNA modification enzymes were purchased from New England Biolabs or Roche Molecular Biochemicals and were used according to the manufacturers' instructions. DNA procedures were carried out according to standard protocols (Sambrook *et al.*, 1989).

### Cloning and PCR amplification of Fg constructs.

*E. coli* strains expressing human Fg A*α*-, B*β*- and *γ*-chains were provided by Professor Susan Lord, Department of Pathology, University of North Carolina at Chapel Hill. A 1878 bp fragment from plasmid p166.9 (Lord, 1985) and a 1236 bp fragment from p253 (Bolyard & Lord, 1988) were subcloned into the plasmid pQE30 (Qiagen) to produce recombinant mature A*α*- and *γ*-chains with N-terminal His tags. Subcloning of the mature recombinant B*β*-chain was described previously (Davis *et al.*, 2001). The recombinant plasmids were transformed into *E. coli* strain JM101 for protein expression.

Segments of the A*α*-chain were amplified by PCR from the plasmid containing the full-length A*α*-chain (residues 1–625 in the common Fg *α*-chain, NCBI accession number AAA17055). Although Fg *α*-chain DNA encodes 625 residues, in plasma the A*α*-chain is only 610 residues long due to a post-translational modification. The oligonucleotide primers used for PCR are listed in Table 1. Restriction sites were incorporated at the 5′ ends of the primers to facilitate directional cloning. PCR amplification was carried out in a DNA thermal cycler (Perkin-Elmer Cetus) with Phusion DNA-polymerase (Bioline). Reactions were carried out with a 30 s denaturation step at 98 °C, a 10 s annealing step (the temperature of which depended on the individual primers) and elongation at 72 °C depending on the length of the PCR product. This standard cycle was repeated 25 times followed by incubation at 72 °C for 10 min. PCR products were purified using the Favourgen Gel/PCR Purification Kit (Favourgen Biotech), cleaved with the appropriate restriction enzyme and cloned into plasmid pQE30.

### Mutagenesis of A*α*-chain of Fg.

Site-directed mutagenesis was carried out on pQE30 carrying the gene encoding A*α*-1–625 using Quickchange (Stratagene). The primers used in the mutagenesis protocol are listed in Table 1. Two mutants were created: A*α*-S317P and A*α*-T322P.

Tandem repeat 5 of A*α*-S317P was made to resemble repeat 3. Repeat 5 of A*α*-T322P resembles repeats 1 and 2, which have proline residues in the middle of their repeat sequences.

### Construction of recombinant *α*-chain mutants.

Sections of the A*α*-chain were deleted by inverse PCR using oligonucleotide primers in Table 1. The forward primers incorporated a complete *Eco*RV site and the reverse primers contained half an *Eco*RV site at their 5′ ends, respectively. The PCR products were digested with *Eco*RV, religated and transformed into *E. coli* XL-1 Blue cells. Amino acid residues D and I were inserted in place of the region of DNA that was deleted from the *α*C-domain.

### Sequencing of recombinant plasmids.

All recombinant plasmids were sequenced by GATC Biotech.

### Expression and purification of recombinant proteins.

Recombinant rClfA 221–559, rClfB 45–542 and rClfB 197–542 were purified by Ni^2+^ affinity chromatography as described previously (O'Connell *et al.*, 1998; Perkins *et al.*, 2001). Purification of the recombinant Fg A*α*-, B*β*- and *γ*-chains, and each of the recombinant A*α*-chain deletion and truncated proteins, was carried out by Ni^2+^ affinity chromatography (Perkins *et al.*, 2001) with the addition of 6 M urea (Sigma) to the bacterial cell suspension prior to lysis.

### SDS-PAGE and Western immunoblotting.

Recombinant proteins were analysed by SDS-PAGE by standard procedures (Laemmli, 1970) on gels containing 10–15 % acrylamide. Gels were stained with Coomassie blue. Human Fg (Calbiochem) was separated by SDS-PAGE and transferred electrophoretically to PVDF Western blotting membranes (Roche Applied Science) by the wet system (Bio-Rad) in Tris/HCl (0.02 M), glycine (0.15 M) and methanol (20 %, v/v). Membranes were blocked for 15 h at 4 °C in 10 % (v/v) non-dry fat milk and then incubated with recombinant ClfA or rClfB (10 μg ml^−1^) for 1 h with shaking. The membranes were washed three times with PBS containing Tween 20 (0.01 %, w/v) and then incubated with polyclonal antisera to ClfA (1 : 2000) (McDevitt *et al.*, 1995) or ClfB (1 : 2000) (McAleese *et al.*, 2001) as appropriate. Horseradish peroxidase (HRP)-labelled goat anti-rabbit IgG (Dako, 1 : 2000) was used to detect bound antibody. Membranes were developed using LumiGLO chemiluminescent substrate (New England BioLabs) according to the manufacturer's instructions and exposed to X-ray film.

### Fibrinogen.

Native human Fg was from Calbiochem. Recombinant Fg A*α*251 contains A*α*-chains truncated at residue 251 but is otherwise identical to normal human Fg. It was purified from CHO cells media (Gorkun *et al.*, 1998). Plasmin digestion of native Fg was performed to isolate fragment D-domains (Rudchenko *et al.*, 1996). Briefly, Fg (70 mg) in Tris-buffered saline (TBS) pH 7.4 was treated with plasmin [0.0015 unit (mg Fg)^−1^] for 6 h at room temperature. The reaction was stopped by the addition of phenylmethylsulfonyl fluoride (Sigma) to 0.5 mM final concentration and dialysed overnight at 4 °C into TBS pH 7.4. The sample was then applied to a MonoQ HP anion-exchange column and eluted with a gradient of 0–1 M NaCl. SDS-PAGE and Western blotting with anti-Fg domain D and anti-Fg domain E polyclonal antibodies (Cambio) showed the D- and E-domains eluting at 250 mM and 350 mM NaCl, respectively.

### Adherence of bacteria to immobilized proteins.

Adherence of *S. aureus* or *L. lactis* to immobilized proteins was performed as described previously (Walsh *et al.*, 2004). Nunc-Immuno MaxiSorb microtitre plates were coated with the protein in carbonate buffer (15 mM Na_2_CO_3_, 35 mM NaHCO_3_, pH 9.6) and incubated overnight at 4 °C. Wells were washed with PBS, BSA (5 mg ml^−1^) was added and the plates were incubated for 2 h at 37 °C. The plates were washed three times with PBS. A bacterial cell suspension (OD_600_ 1.0 in PBS) was added (100 μl per well) and the plates were incubated for 1 h at room temperature. Plates were washed three times with PBS and bound cells were fixed with formaldehyde (25 %, v/v) for 30 min and stained with crystal violet (0.5 %, v/v 100 μl per well) for 1 min. Following three washes with PBS, acetic acid (5 %, v/v) was added (100 μl per well) for 10 min at room temperature. The absorbance was measured at 570 nm in an ELISA plate reader (Labsystems Multiskan Plus). Inhibition of bacterial adherence by rClfB 197–542 was performed as described by Perkins *et al.* (2001).

### Binding of rClfB 45–542 to immobilized proteins.

ELISA plates were coated with the appropriate protein in carbonate buffer overnight at 4 °C. Wells were washed twice with PBS and incubated at 37 °C with BSA in PBS for 2 h at 37 °C. They were then washed with PBS and varying concentrations of rClfB 45–542 in PBS with Tween 20 (0.01 %, w/v) were added. The plates were then incubated for 1 h at room temperature. Any unbound protein was removed by washing with PBS, and plates were incubated with rabbit anti-ClfB 45–452 antibodies for 1 h at room temperature. Wells were washed and HRP-labelled goat anti-rabbit IgG (1 : 2000) was added for 1 h at room temperature. After washing, 1 mg ml^−1^ tetramethylbenzidine chromogenic substrate and 0.006 % (v/v) H_2_O_2_ in 0.05 M phosphate citrate buffer pH 5.0) was added (100 μl per well) and plates developed for 10 min in the dark. The reaction was stopped by the addition of 2 M H_2_SO_4_ (50 μl per well), and plates were read at 450 nm.

To check that native and mutant Fg A*α*251 were coating the ELISA plates efficiently, HRP-labelled anti-human Fg antibody (Dako 1 : 4000) was added to the plates following the initial blocking step. The plates were incubated at room temperature for 1 h with shaking. After washing with PBS, bound antibody was detected by adding tetramethylbenzidine and H_2_SO_4_ and reading the absorbance at 450 nm.

## RESULTS

### ClfB binds to the *α*-chain of human Fg

**Fig. 2. f2:**
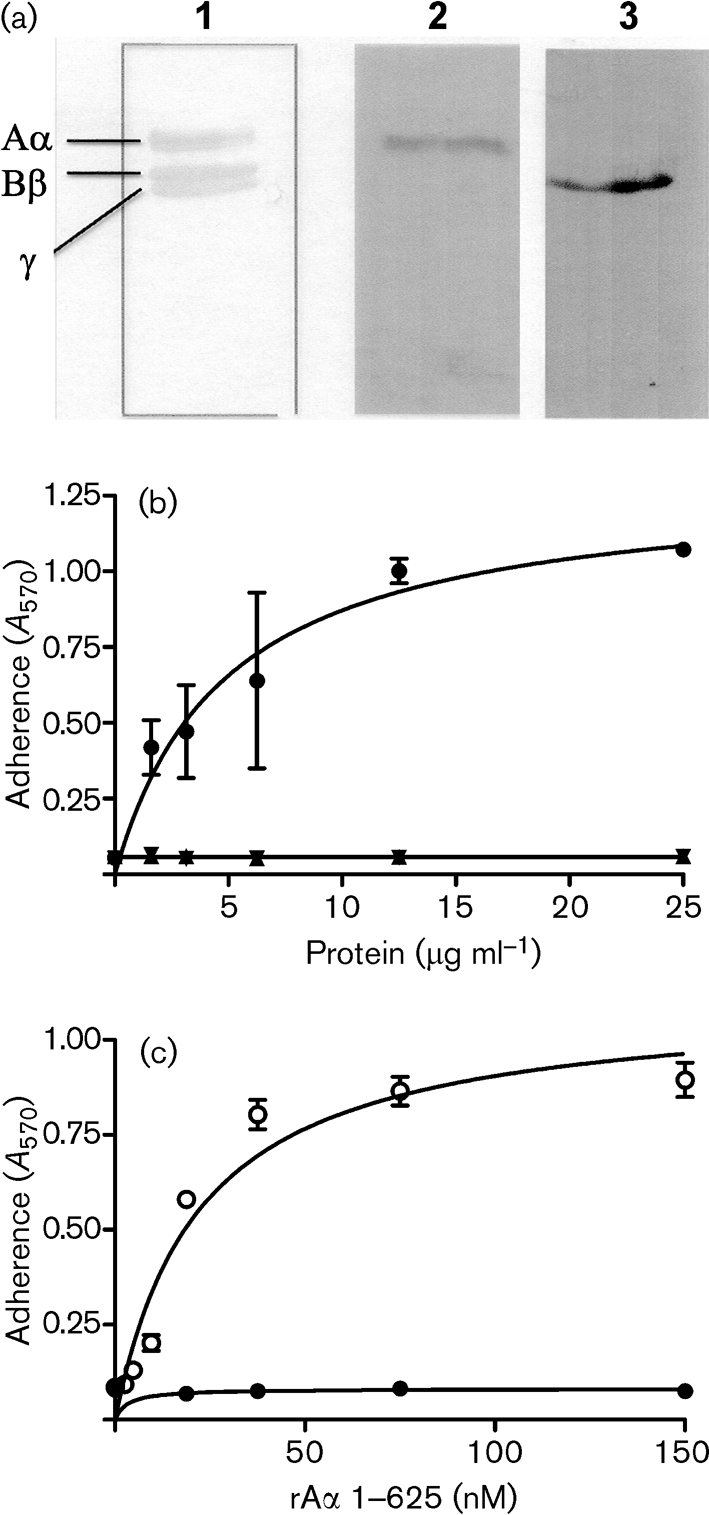
ClfB binds to Fg A*α*-chain. (a) Ligand affinity blotting. Human Fg was fractionated by SDS-PAGE and stained with Coomassie blue (lane 1), or transferred to a PVDF-membrane and probed with rClfB 45–542 (lane 2) or rClfA 220–559 (lane 3). Binding of rClf proteins was detected with anti-Clf polyclonal antisera. (b) Adherence of *S. aureus* Newman ClfA^−^ to immobilized recombinant A*α*- (•), B*β*- (▴) or *γ*- (▾) chains of Fg. (c) Adherence of *S. aureus* Newman ClfA^−^ (○) and Newman ClfA^−^ ClfB^−^ (•) to immobilized recombinant A*α*-chain of Fg. Cells were grown to exponential phase and suspensions (1×10^8^ c.f.u.) were added to wells. Adherent bacteria were detected by crystal violet staining. The experiment was performed twice with similar results and values represent means±sd of triplicate wells.

Native whole human Fg was separated into individual A*α*-, B*β*- and *γ*-chains by denaturing SDS-PAGE, transferred to PVDF membranes and probed with recombinant (r) ClfB region A-domain (rClfB 45–542). Binding of rClfB was detected with anti-ClfB region A antibodies. The rClfB protein bound specifically to the A*α*-chain (Fig. 2a[Fig f2]). In contrast, rClfA region A-domain rClfA 40–559 reacted with the *γ*-chain of human Fg (Fig. 2a[Fig f2]), confirming previous reports (McDevitt *et al.*, 1997).

**Fig. 3. f3:**
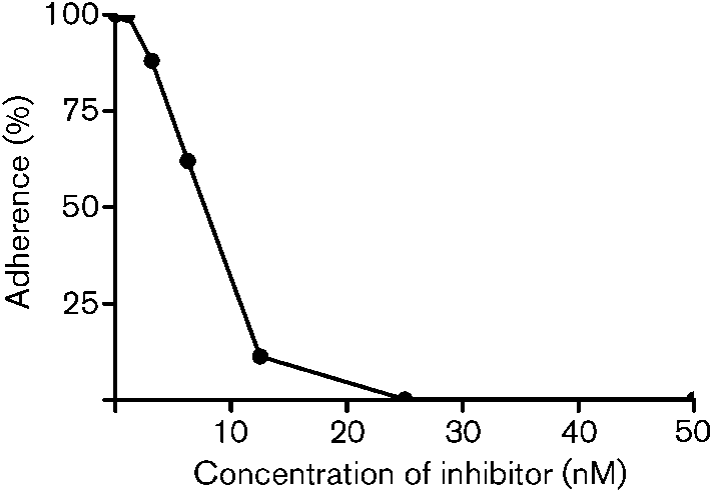
Inhibition of *S. aureus* Newman ClfA^−^ adherence to recombinant A*α*-chain by rClfB 197–542. ELISA plates coated with Fg rA*α*-chain 1–625 were incubated with increasing concentrations of rClfB 197–542 for 1 h at room temperature. Newman ClfA^−^ cells grown to exponential phase (1×10^8^ c.f.u.) were added to wells and adherent bacteria were detected by crystal violet staining. The values represent means of triplicate wells.

Purified recombinant Fg A*α*-, B*β*- and *γ*- chains were immobilized and tested for their ability to support the adherence of ClfB-expressing *S. aureus* cells. The ability of *S. aureus* Newman cells defective in ClfA to adhere to Fg is exclusively due to expression of ClfB. Newman does not express the bifunctional fibronectin- and Fg-binding proteins FnBPA and FnBPB (Wann *et al.*, 2000). Newman *clfA* adhered to recombinant A*α*-chain (rA*α* 1–625) in a dose-dependent and saturable manner but did not adhere to either the recombinant B*β*-chain or the *γ*-chain (Fig. 2b[Fig f2]). The double *clfA clfB* mutant did not adhere to Fg or the A*α*-chain (Fig. 2c[Fig f2]). These data clearly show that ClfB binds only to the A*α*-chain of Fg and not to the B*β*-chain as was reported previously (Ni Eidhin *et al.*, 1998). A truncated but functional form of rClfB 197–542 (Perkins *et al.*, 2001) inhibited adherence of Newman *clfB* to rA*α* 1–625 in a dose-dependent manner (Fig. 3), indicating that recombinant ClfB and ClfB expressed by *S. aureus* compete for the same binding site(s).

### ClfB binds to a site within the *α*C-domain of human Fg

**Fig. 4. f4:**
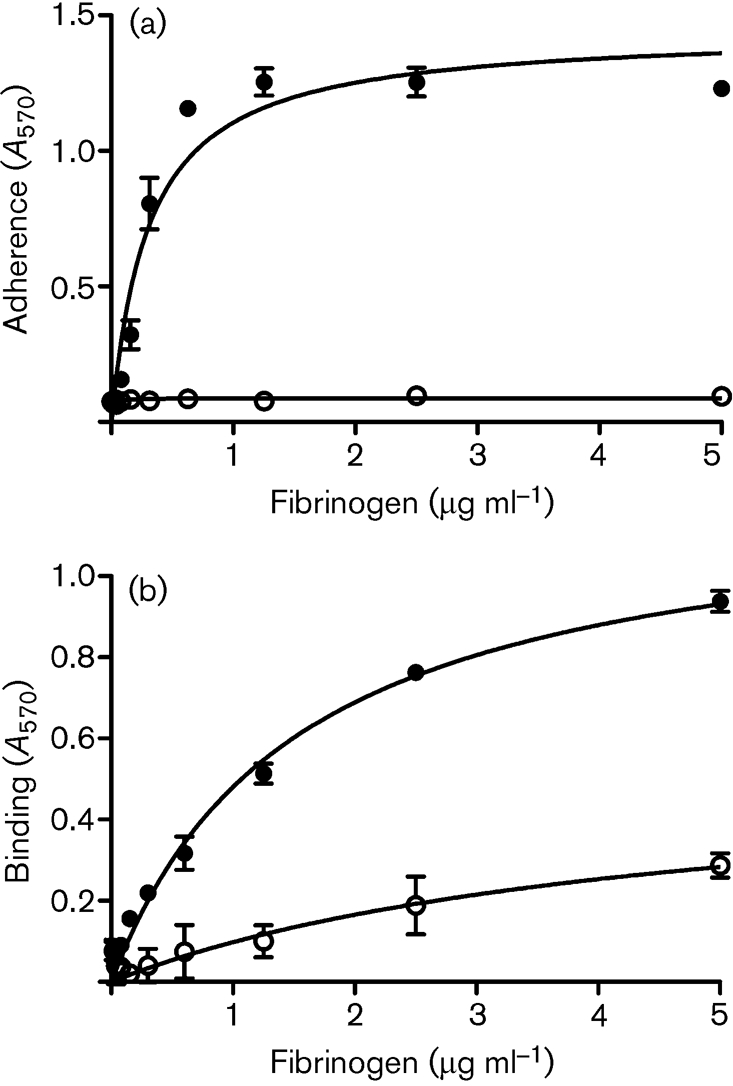
Binding to native and mutant Fg. (a) Adherence of *S. aureus* Newman ClfA^−^ cells to immobilized native Fg (•), or mutant Fg A*α*251 (○). Exponential-phase cells (1×10^8^ c.f.u.) were added to wells and adherent bacteria were detected by crystal violet staining. (b) Binding of rClfB 45–542 to native Fg (•), or mutant Fg A*α*251 (○). The experiment was performed twice with similar results and the values represent means±sd of triplicate wells.

Mutant recombinant human Fg with a deletion of residues 252–625 comprising the C terminus of the A*α*-chain (Fg A*α*251) was purified from CHO cells (Gorkun *et al.*, 1998). Native human Fg and Fg A*α*251 were coated onto the wells of a microtitre dish and tested for their ability to support adherence of *S. aureus* cells and binding by rClfB protein. *S. aureus* Newman *clfA* adhered strongly to native Fg but did not bind to Fg A*α*251 (Fig. 4a[Fig f4]). Also rClfB 45–542 bound very weakly to Fg A*α*251 in an ELISA-based assay (Fig. 4b[Fig f4]). Plasmin treatment of Fg cleaves the protein into an E-fragment, two D-fragments and two *α*C-domains. Purified D- and E-domains of Fg did not inhibit the adherence of *S. aureus* cells to Fg (data not shown). These data suggest that the binding site for ClfB in Fg lies in the C-terminal region of the A*α*-chain of Fg between residues T251 and V610.

**Fig. 5. f5:**
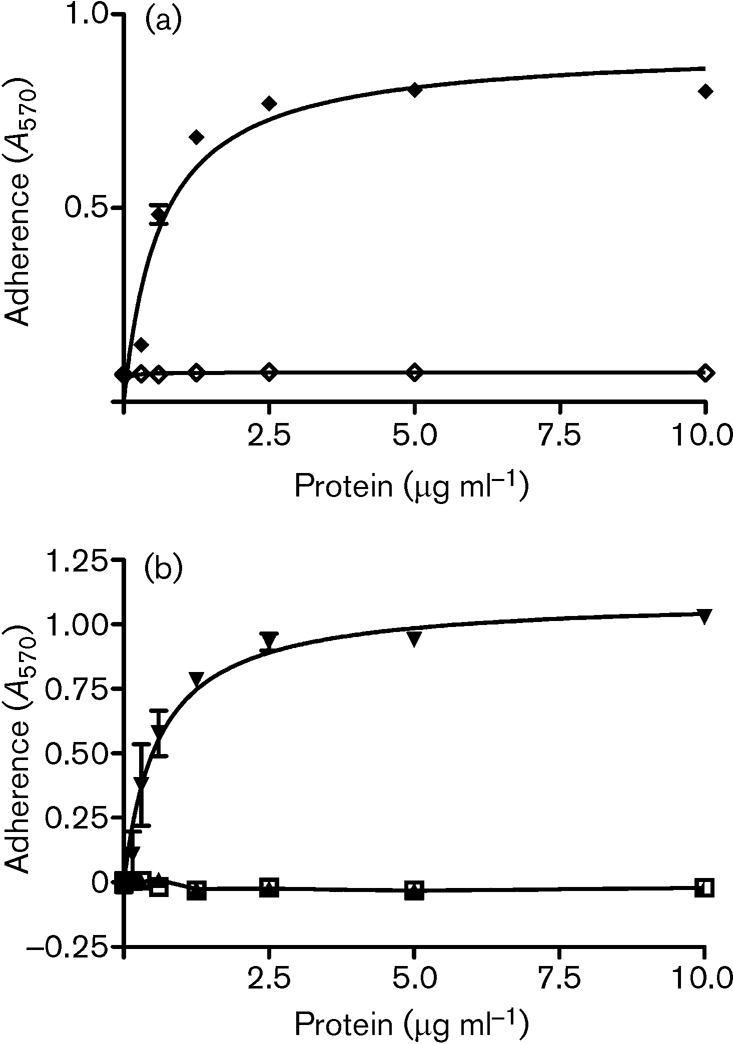
Localization of the ClfB-binding site in Fg using recombinant A*α*-chain truncates. (a) Adherence of *S. aureus* Newman ClfA^−^ to immobilized recombinant Fg A*α*-chain truncates, rA*α* 232–625 (⧫) and rA*α* 1–283 (□). (b) Adherence of Newman ClfA^−^ to rA*α* 367–574 (□), rA*α* 367–625 (▴) and rA*α* 315–574 (▾). Increasing concentrations of recombinant *α*-chain constructs were immobilized on ELISA plates and exponential-phase cells (1×10^8^ c.f.u.) were added to the wells. Adherent bacteria were detected by crystal violet staining. The values represent the means±sd of triplicate wells.

To investigate further the binding site for ClfB within the C terminus of the A*α*-chain, a series of truncated forms of the recombinant *α*-chain were expressed (Fig. 5a[Fig f5]). The proteins were purified, immobilized in ELISA wells and tested for their ability to support the adherence of *S. aureus* Newman *clfA*. Bacteria adhered to the protein corresponding to the C-terminal half of the A*α*-chain (rA*α* 232–625) but did not adhere to the N-terminal region (rA*α* 1–283, Fig. 5a[Fig f5]), confirming that the ClfB-binding site in the Fg *α*-chain is in the C-terminal domain. The C-terminal *α*-chain construct (rA*α* 232–625) was further truncated in order to locate the binding site for ClfB (Fig. 5b[Fig f5]). The truncated proteins were tested for their ability to support the adherence of ClfB^+^ *S. aureus* cells. The smallest truncate that supported binding of ClfB was rA*α* 315–574, whereas rA*α* 367–625 did not, suggesting that ClfB binds to a site between residues W315 and W367 in the *α*C-domain.

### ClfB binds to repeat region 5 within the flexible connector region of the A*α*-chain

**Fig. 6. f6:**
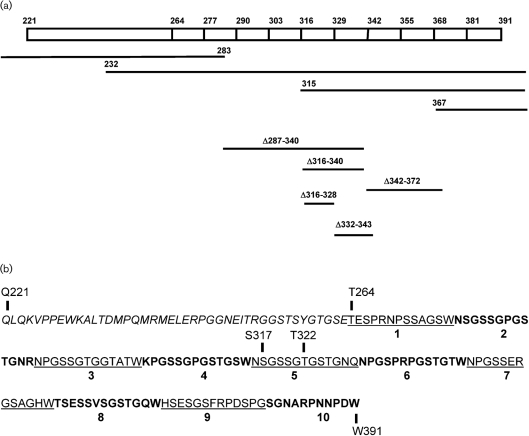
Schematic diagram showing the organization of the *α*C-domain of human Fg. (a) The unordered NH_2_-terminal half (residues 221–391) is composed of a 43 amino acid segment followed by ten 13-residue tandem repeats. The recombinant A*α*-chain proteins used in this study are shown by lines and correspond to truncated or deleted variants. (b) Amino acid sequence of the N-terminal part of the *α*C-domain. The ten tandem repeats begin at T264 and are shown in alternating normal and bold type.

**Fig. 7. f7:**
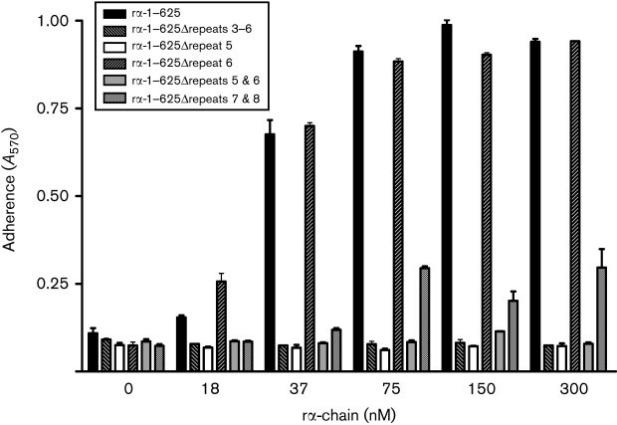
Localization of the ClfB-binding site in the tandem repeat region of the *α*C-domain of Fg using recombinant A*α*-chain deletions. Increasing concentrations of recombinant *α*-chain constructs were immobilized on ELISA plates. Exponential-phase Newman ClfA^−^ cells (1×10^8^ c.f.u.) were added to the wells and adherent bacteria were detected by staining with crystal violet. Error bars represent standard deviations for three replicates. The experiment was performed twice with similar results.

The structural organization of the A*α* C-terminal domain (residues 221–610) of human Fg has not yet been fully established. It has been shown that each *α*C-domain consists of two structurally distinct regions, a compact C-terminal half connected to the rest of the molecule via an unordered NH_2_-terminal connector region (Fig. 1) (Burton *et al.*, 2006; Tsurupa *et al.*, 2002). In human Fg the flexible connector region starts with a 43-residue segment followed by ten 13-residue tandem repeats (Fig. 6) (Tsurupa *et al.*, 2002). Using rA*α*-chain truncates we located the binding site for ClfB to between residues W315 and W367 (Fig. 5), corresponding to tandem repeats 5–8. Deletion mutants that had lost one or more of the tandem repeats were created in the plasmid expressing A*α* 1–625. The ability of *S. aureus* ClfB^+^ cells to adhere to the recombinant mutant proteins was investigated (Fig. 7). *S. aureus* cells adhered in a dose-dependent and saturable manner both to the wild-type recombinant A*α*-chain and to an A*α*-chain construct that contained a deletion of most of repeat 6 (A*α* 1–625 Δ332–343). Cells also adhered to a construct with deletion of repeats 7 and 8 (A*α* 1–625 Δ342–372), although the binding appeared to be weaker (Fig. 7). However, ELISA with anti-Fg antibodies showed that rA*α* 1–625 Δ342–372 coated the plates poorly compared to all of the other proteins (data not shown), which might explain the lower adherence. Crucially, a deletion lacking repeat 5 (rA*α* 1–625 Δ316–328) failed to support adherence, suggesting that the ClfB-binding site is located in this region of the *α*-chain of Fg.

### Altering residues in repeat 5 of the A*α*-chain disrupts binding to ClfB

**Fig. 8. f8:**
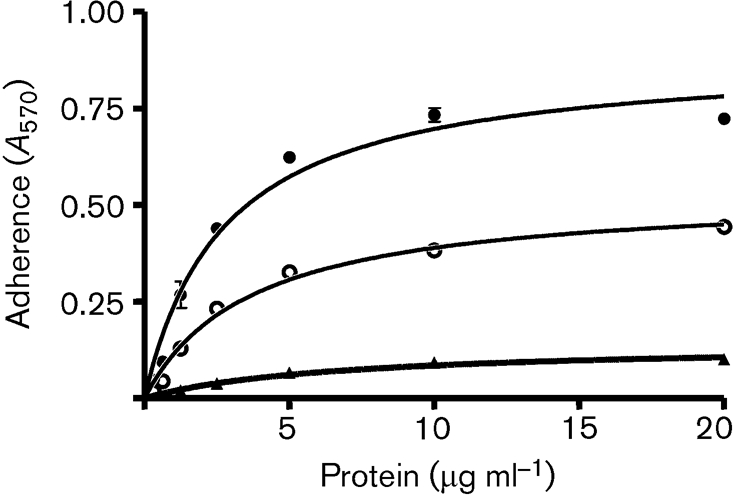
Adherence of *S. aureus* Newman ClfA^−^ cells to immobilized recombinant Fg A*α*- (•), A*α*S317P- (○) and A*α*-T322P- (▴) chains. Increasing concentrations of recombinant *α*-chain constructs were immobilized on ELISA plates. Exponential-phase Newman ClfA^−^ cells (1×10^8^ c.f.u.) were added to the wells and adherent bacteria were detected by staining with crystal violet. Bars represent standard deviations for three replicates. The experiment was performed twice with similar results.

The possibility that an additional ClfB-binding site occurs in repeats 1–3 (residues 264–302) was investigated by isolating two proline substitutions within repeat 5 of rFg A*α* 1–625. Purifed recombinant A*α* 1–625 chain and two mutants (S317P and T322P) were tested for their ability to support adherence of Newman *clfA*. ELISA with anti-6xHis antibody (Roche) showed that each protein coated the microtitre plates equally well (data not shown). Newman *clfA* bound to the wild-type A*α*-chain in a dose-dependent and saturable manner (Fig. 8). Newman *clfA* was not able to adhere to the A*α*-T322P mutant, while adherence to the A*α*-S317P mutant was reduced. The inability of A*α*-T322P to support binding to ClfB suggests that there is a single binding site for ClfB in the *α*-chain of Fg located in repeat 5.

### Expression of ClfB mutants by *L. lactis*

**Fig. 9. f9:**
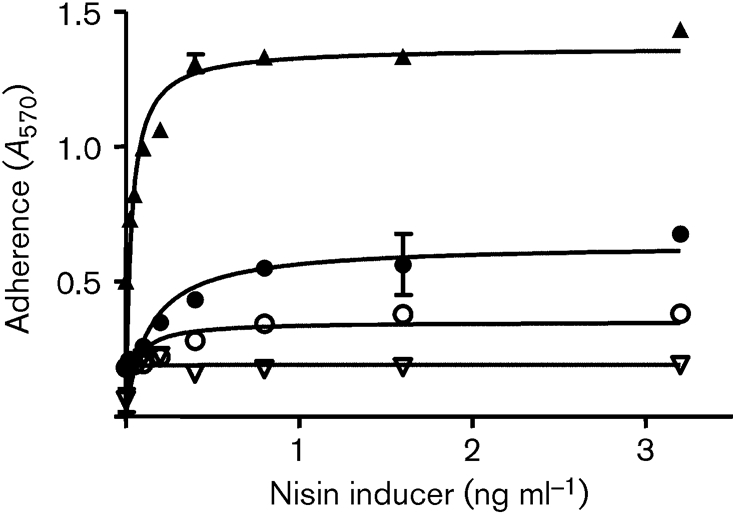
Fg binding by *L. lactis* ClfB^+^ and *L. lactis* ClfB mutants. *L. lactis* ClfB^+^ (▴), *L. lactis* ClfB Q235A (○), *L. lactis* ClfB N526A (•) and *L. lactis* pNZ8037 (▿) were induced with nisin and grown to stationary phase. Adherence of washed cultures to ELISA plates coated with Fg (10 μg ml^−1^) was assessed by crystal violet staining; the values represent the means±sd of triplicate wells.

In order to investigate if the trench located between domains N2 and N3 of ClfB is important in binding to the Fg A*α*-chain, several residues with side chains that were predicted to be located close to or within the trench were converted to alanine and expressed on the surface of *L. lactis* NZ9800 from the nisin-inducible vector pNZ8037. *L. lactis-*expressing mutants Q235A and N256A were defective in adherence to immobilized Fg (Fig. 9) and CK10 (data not shown) compared to the wild-type control. Western immunoblotting indicated that the proteins were expressed at the same level as the wild-type and were intact. This suggests that CK10 and Fg likely bind to the same region of ClfB.

## DISCUSSION

Many aspects of haemostasis and wound healing involve the *α*C-domains of Fg playing a central role. The two self-interacting *α*C-domains are formed by the C-terminal two-thirds of the two A*α*-chains (residues 220–610) (Burton *et al.*, 2006; Medved *et al.*, 1983). Compared to the rest of the Fg molecule, the *α*C-domains are very sensitive to proteases and are readily cleaved into smaller fragments (Doolittle, 1984; Henschen & MCDonagh, 1986; Weisel & Medved, 2001), and they are the first portions of fibrin to be removed upon fibrinolysis (Tsurupa & Medved, 2001).

This paper presents data that define the binding site in Fg for ClfB as being located in repeat 5 of the flexible region of the A*α*-chain. Only the A*α*-chain and not the B*β*- or *γ*-chains could support binding of ClfB. Fg with a deletion of the entire *α*C region and a recombinant A*α*-chain mutant lacking the C-terminal *α*C region did not support binding. A series of recombinant truncated proteins narrowed down a binding domain to repeat 5 located between residues 316 and 328.

In human Fg each individual tandem repeat is composed of 13 amino acids (Fig. 6). Up to eight residues in the repeats are glycine or serine. Repeat 5 (NSGSSGTGSTGNQ) may form a loop similar to the Tyr-(Gly/Ser)*_n_* Ω loops present in the tail region of CK10, to which ClfB also binds (Walsh *et al.*, 2004). Since the repeats that apparently do not support ClfB binding contain proline and/or arginine residues (Fig. 6), it is possible that these residues interfere with the potential of ClfB to bind to other parts of the repeat region. To examine this, two mutants in repeat 5 mimicking the presence of proline residues located in other putative non-ClfB-binding repeats were isolated in a recombinant Fg *α*-chain construct that contained each of repeats 1–8. The S317P mutant had reduced affinity for ClfB whereas the T322P mutant was unable to bind ClfB. This suggests that repeat 5 is the only site in the Fg *α*-chain that ClFB binds to, and that T322 is crucially important for this. The presence of a P in the centre of the putative Ω loops in other repeats, in particular repeat 2, might explain their apparent inability to support binding in the T322 mutant. The S317P substitution creates a sequence in repeat 5 that resembles repeat 3. It is unclear why wild-type repeat 3 did not support reduced ClfB binding in the S317 mutant similar to the T322P substitution in repeat 5. Perhaps the sequences at the C terminus of repeat 3 that differ from repeat 5 are responsible.

This study suggests that Fg and CK10 have the same or overlapping binding sites on ClfB (Walsh *et al.*, 2004) and that the mechanism of ClfB binding to Fg is likely to be similar to K10 binding. Amino acid substitution mutants Q235A and N256A located in the putative binding trench in ClfB between domains N2 and N3 were defective in binding to both Fg and CK10.

A common theme is emerging concerning the nature of ligands recognized by surface proteins of staphylococci of the Clf-Sdr family. Each binds flexible unfolded peptides, with ClfA and FnBPA binding to the short flexible *γ*-chain C-terminal peptide that protrudes from domain D of Fg (McDevitt *et al.*, 1997) while SdrG binds to peptides at the N-terminal end of the *β*-chain that extend from domain E (Davis *et al.*, 2001; Ponnuraj *et al.*, 2003). ClfB binds to glycine- and serine-rich loops at the C terminus of CK10 (Walsh *et al.*, 2004) and this study reveals that ClfB also binds to the flexible connector region of the *α*C region of Fg. It is logical to postulate that each of these MSCRAMMs binds their ligand(s) by the dock latch and lock mechanism (Ponnuraj *et al.*, 2003) but this can only be shown for certain by solving the X-ray crystal structure of the MSCRAMM with the ligands bound.

## Figures and Tables

**Table 1. t1:** Synthetic oligonucleotides used to amplify A*α*-chain fragments and for site-directed and deletion mutants of the *α*-chain Restriction endonuclease sites are underlined. The nucleotides in boldface indicate the location of the desired mutation. F corresponds to forward primer; R corresponds to reverse primer.

**Expression constructs**	**Primer name**	**Primer sequence**
Truncated proteins	A*α*-1F	CGG GGA TCC GCA GAT AGT GGT GAA GGT G
	A*α*-232F	GAC GGA TCC TTA ACA GAC ATG CCG CAG ATG AG
	A*α*-315F	CGG GGA TCC TGG AAC TCT GGG AGC TCT GG
	A*α*-367F	CGG GGA TCC TGG CAC TCT GAA TCT GGA AG
	A*α*-283R	CGG AAG CTT AGG TCC AGA GCT CCC AGA GTT C
	A*α*-574R	CGG AAG CTT TTA GTC TCC TCT GTT GTA ACT CGT G
	A*α*-625R	CGG AAG CTT TTA GGG GGA CAG GGA AGC CTT C
Deletion mutagenesis	A*α*-329F	GCG CTG ATA TCG GAA ACC AAA ACC CTG GGA G
	A*α*-341F	GCG CTG ATA TCG GTA GTA CCG GAA CCT GG
	A*α*-344F	GCG CTG ATA TCT GGA ATC CTG GCA GCT CTG
	A*α*-373F	CGC TGA TAT CGG AAG TTT TAG GCC AGA TAG
	A*α*-286R	ATC TCG GTT TCC AGT ACT TCC AG
	A*α*-315R	ATC CCA GCT TCC AGT ACT TCC AG
	A*α*-331R	ATC GTC CCC AGG GTT TTG GTT CTT
	A*α*-341R	ATC CCA GGT TCC GGT ACT ACC AG
Site-directed mutagenesis	A*α*-S317PF	GCTGGAAC**C**CTGGGAGCTCTGGAAC
	A*α*-S317PR	GTTCCAGAGCTCCCAG**G**GTTCCAGC
	A*α*-T322PF	TGGGAGCTCTGGA**C**CTGGAAGTACTGGAAAC
	A*α*-T322PR	GTTTCCAGTACTTCCAG**G**TCCAGAGCTCCCA
ClfB mutant	N526F	GTT GGA ATA ATG AG**G CT**G TTG TAC GTT ATG GTG G
	N526R	CCA CCA TAA CGT ACA AC**A GC**C TCA TTA TTC CAA C

## Abbreviations

- CK10, cytokeratin 10
- Fg, fibrinogen
- HRP, horseradish peroxidase
- MSCRAMMs, microbial surface components recognizing adhesive matrix molecules
